# Supplementary material for: Stable single platinum atoms trapped in sub-nanometer cavities in 12CaO·7Al2O3 for chemoselective hydrogenation of nitroarenes
Source: Nat Commun. 2020 Feb 24;11:1020. doi: 10.1038/s41467-019-14216-9 (PMC7039943; doi:10.1038/s41467-019-14216-9)
Supplement: Supplementary file 1 — Supplementary Information [file 41467_2019_14216_MOESM1_ESM.pdf]

Supplementary Information

Stable single platinum atoms trapped in sub-nanometer cavities in $12CaO \cdot 7Al_2O_3$ for chemoselective hydrogenation of nitroarenes

Ye et al.

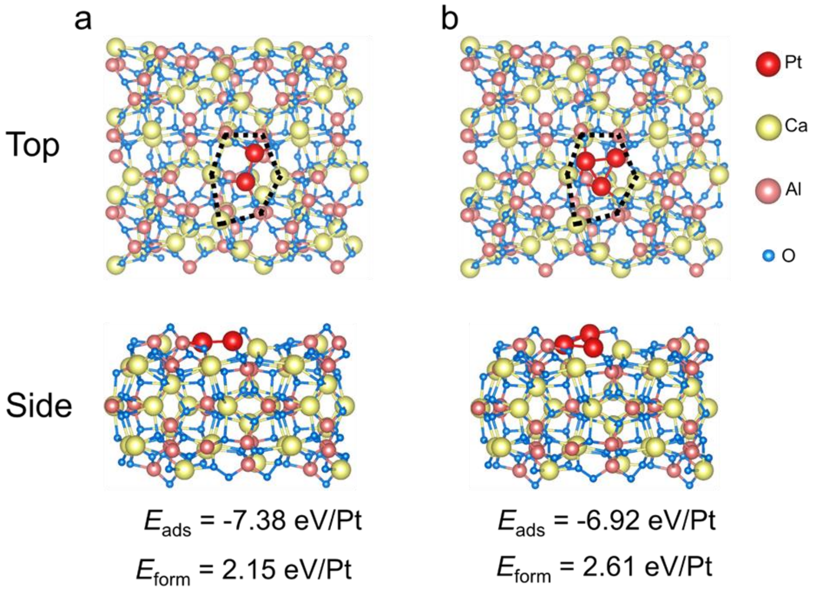

Supplementary Fig. 1 DFT optimized adsorption models of Pt dimer and trimer on the (001) surface of C12A7. The adsorption energy of Pt dimer and trimer is -7.38 eV/Pt and -6.92/Pt eV respectively. Meanwhile, the dimerization and trimerization energy per Pt atoms is also calculated from the Pt ensemble effect, showing 2.15 eV/Pt and 2.61 eV/Pt respectively, which are prohibitively high so that the dimerization and trimerization can hardly occur.

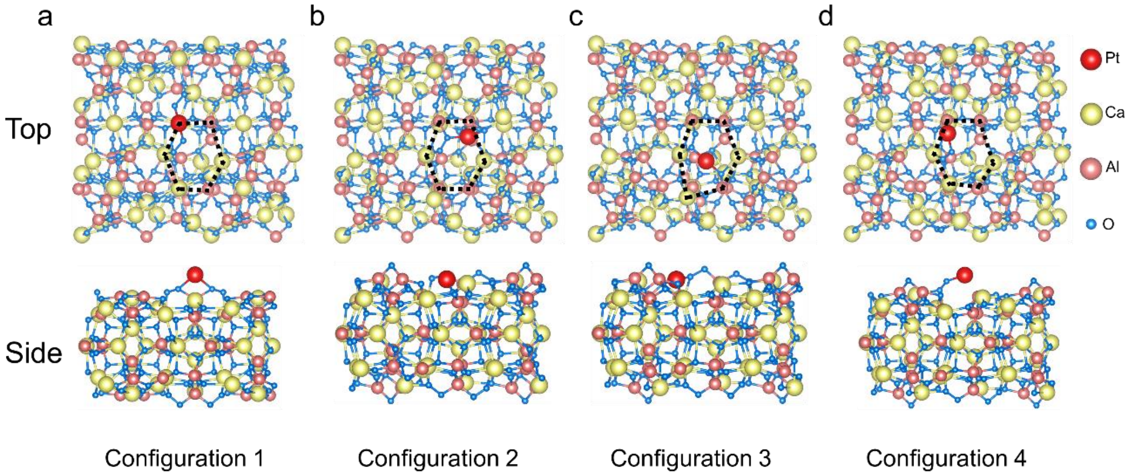

Supplementary Fig. 2 DFT optimized adsorption models of Pt single atoms in C12A7, showing the four identified stable positions of Pt atoms. Two (1 and 4) are located slightly above the Ca-Al-O cavity in the surface of C12A7, and another two (2 and 3) are found to be buried in the Ca-Al-O cavity of C12A7. The difference between configuration 2 and configuration 3 is the Pt location in the cavity. In configuration 2, Pt is restricted as the cavity wall by two oxygen ions, so it is located at the edge of the cavity. While, in configuration 2, the Pt single atom was trapped in the middle of a cavity by two exposed oxygen ions, which is the most stable adsorption models.

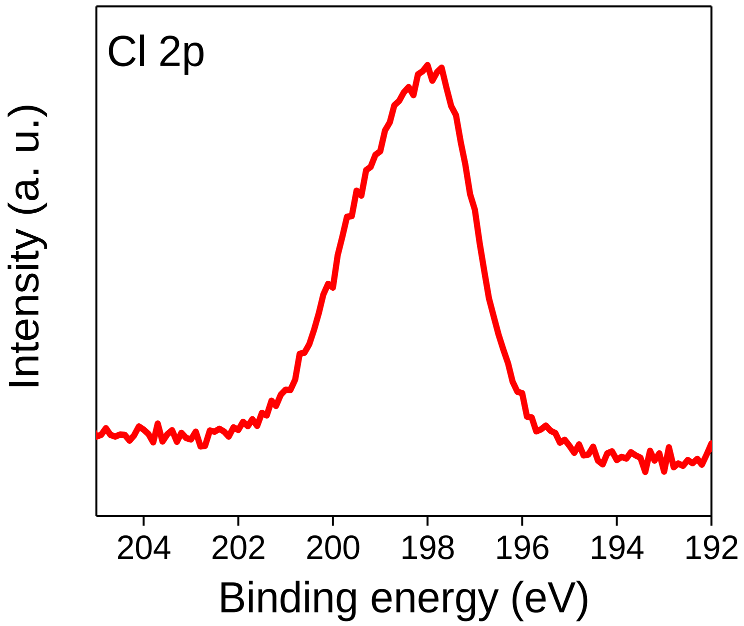

Supplementary Fig. 3 XPS Cl 2p spectrum for the as-prepared 0.1Pt/C12A7 catalyst.

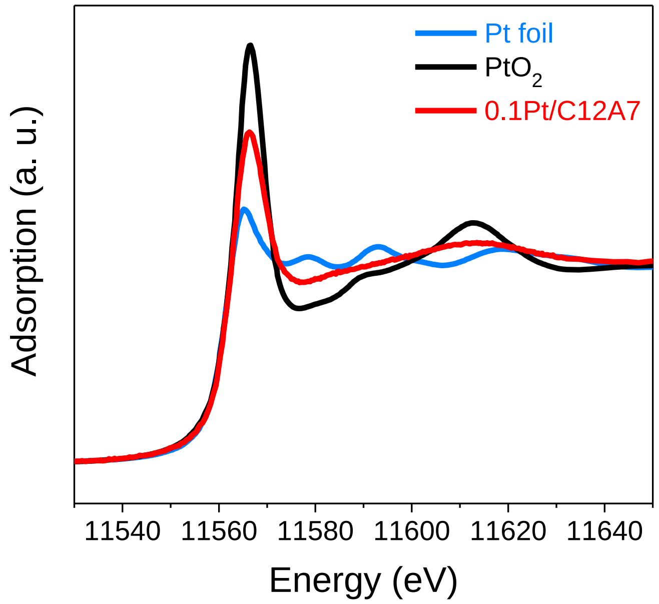

Supplementary Fig. 4 The normalized XANES spectra at the Pt L3-edge of the 0.1Pt/C12A7, PtO_2 and Pt foil samples.

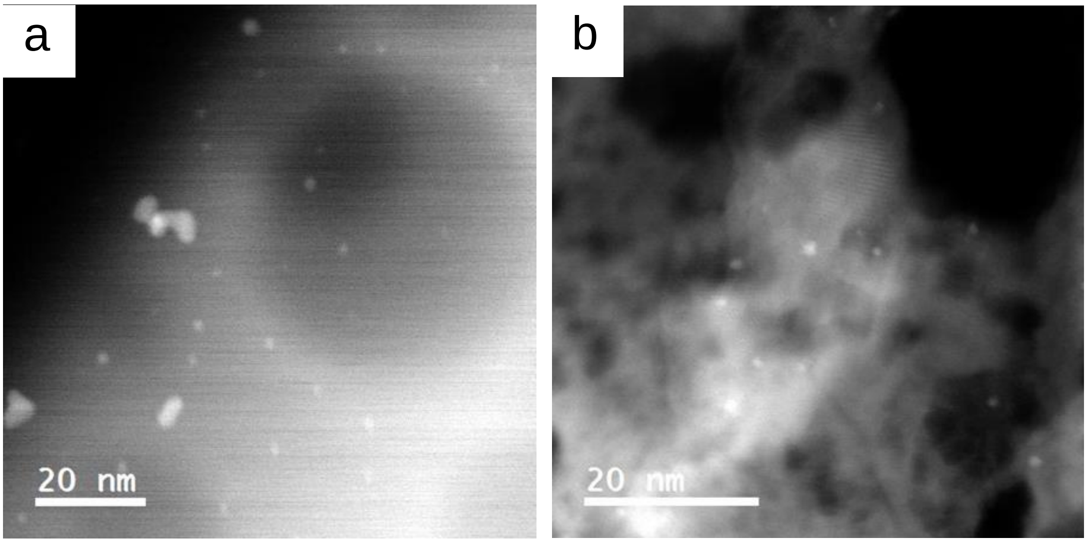

Supplementary Fig. 5 HAADF-STEM images of a) 0.1Pt/Al_2O_3 and b) 0.1Pt/CaO respectively.

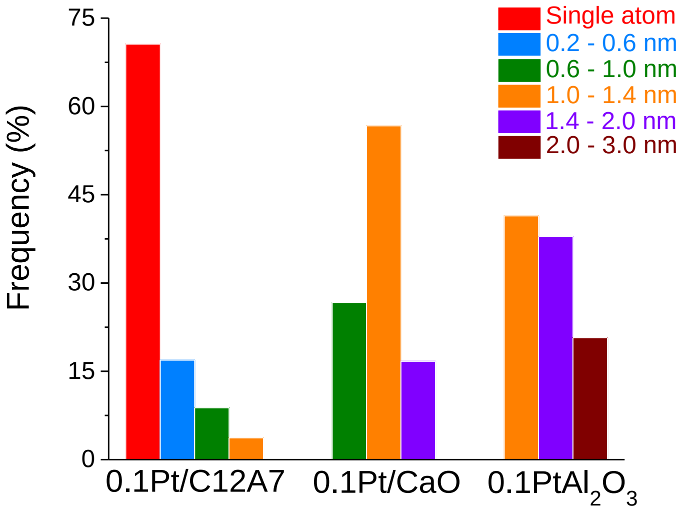

Supplementary Fig. 6 Histogram of size distributions of Pt species on different supports including C12A7, Al_2O_3 and CaO (summarized from HAADF-STEM images).

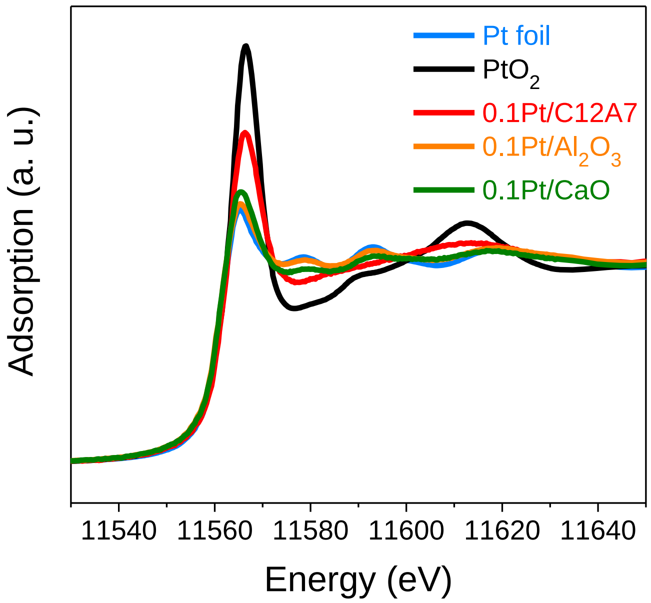

Supplementary Fig. 7 The normalized XANES spectra at the Pt L3-edge of Pt species on C12A7, Al_2O_3 and CaO under similar preparation conditions.

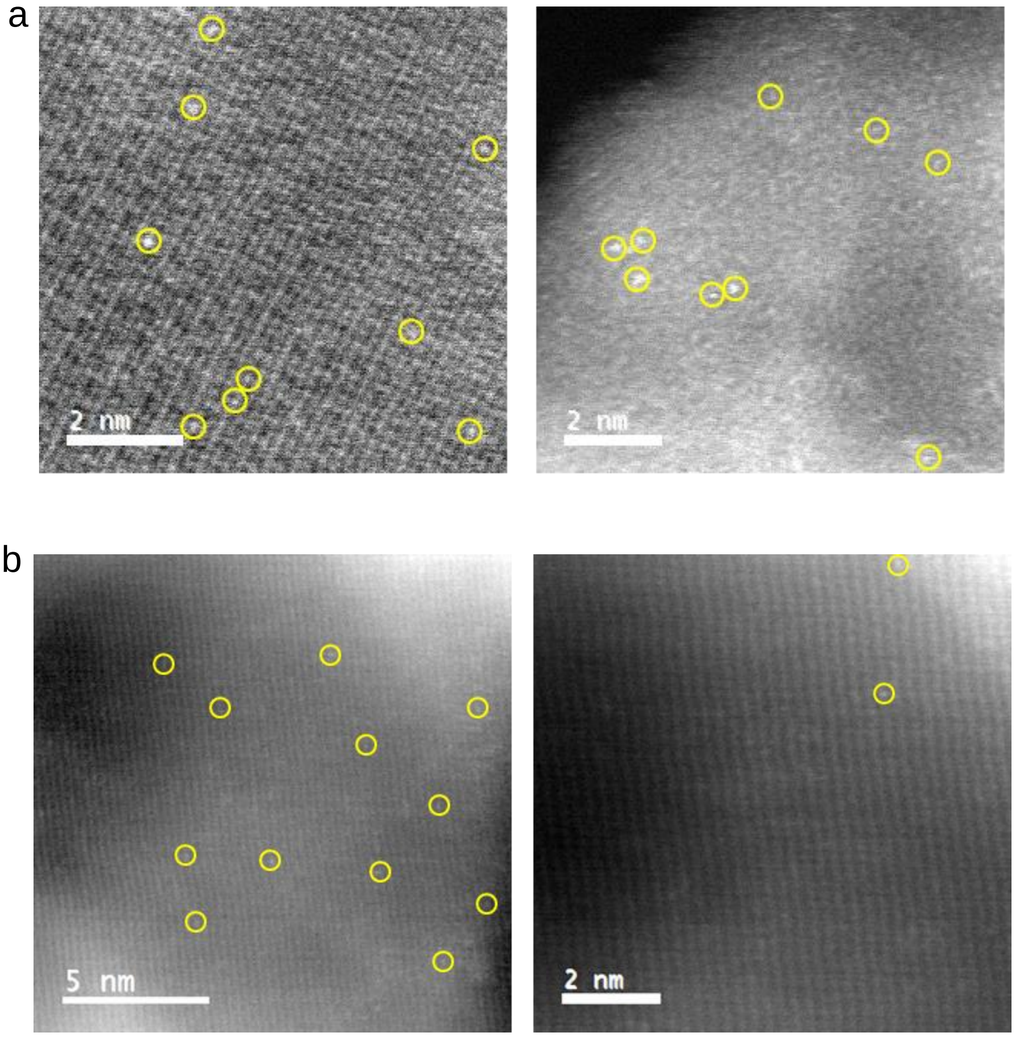

Supplementary Fig. 8 HAADF-STEM images of (a) 0.1Ru/C12A7 and (b) 0.1Rh/C12A7 single-atom structures. Single atoms marked in yellow circles dispersed on the C12A7 support.

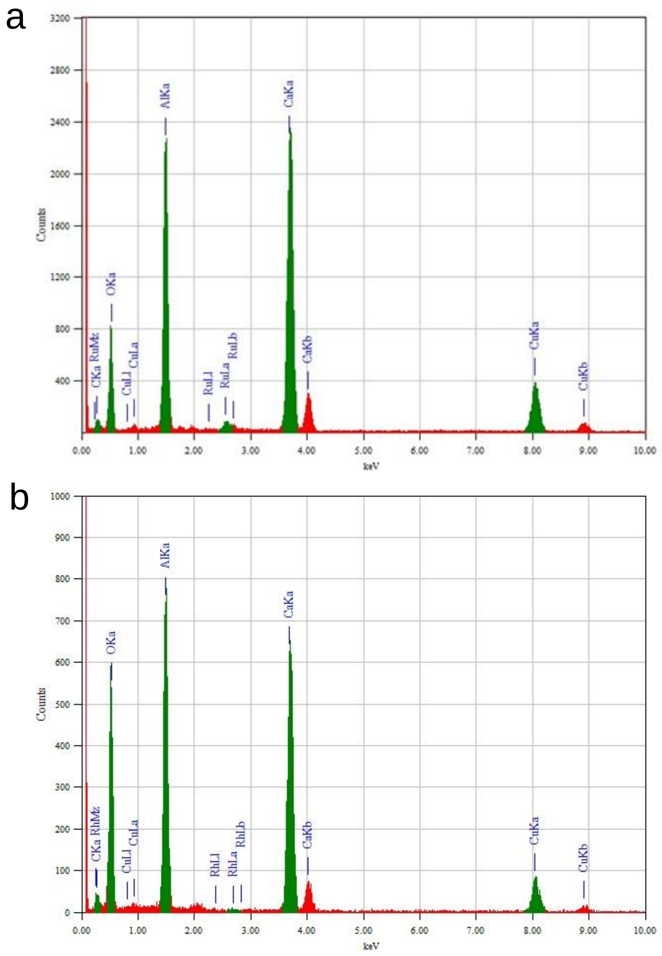

Supplementary Fig. 9 EDX spectra of (a) 0.1Ru/C12A7 and (b) 0.1Rh/C12A7 single-atom structures. Ru can be detect from the EDX spectrum of 0.1Ru/C12A7 (a) and almost no Rh peaks observed in the EDS pattern of 0.1Rh/C12A7, which may due to the detect limit for Rh signals.

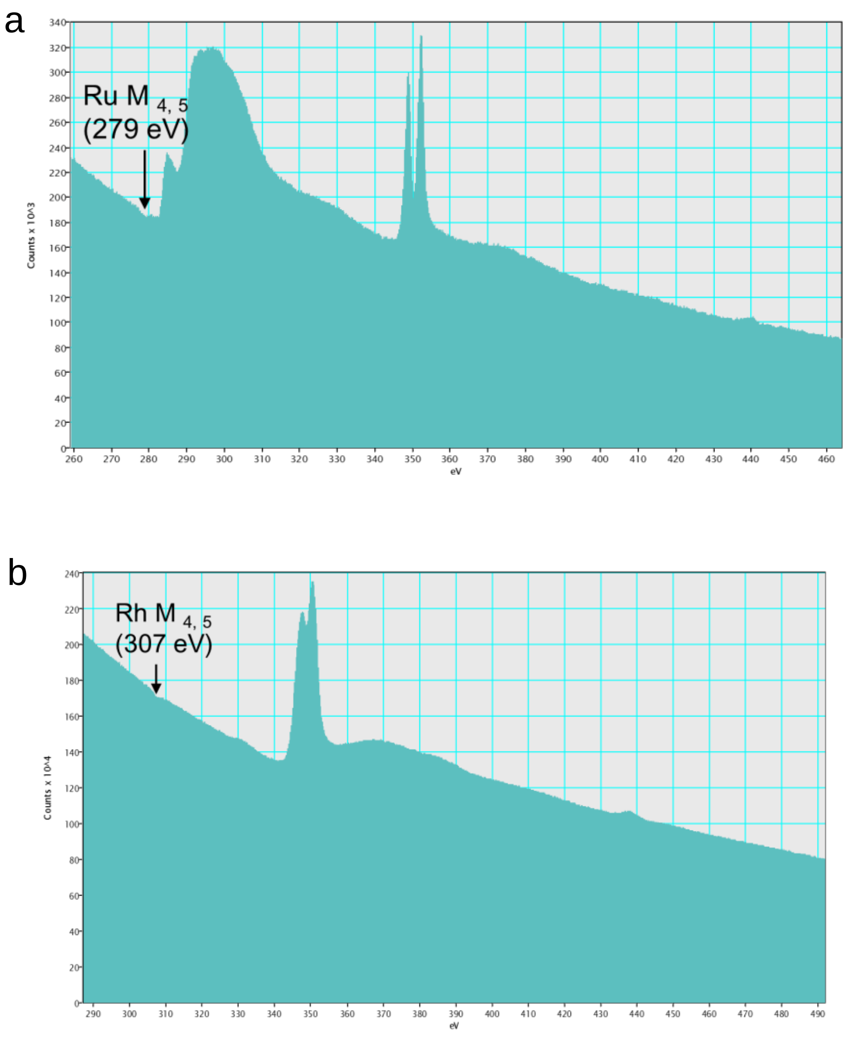

Supplementary Fig. 10 EELS spectra of (a) 0.1Ru/C12A7 and (b) 0.1Rh/C12A7 single-atom structures. The Ru and Rh atoms are identified by the Ru $M_{4,5}$ edge (at 279 eV) and Rh $M_{4,5}$ edge edge (at 307 eV) in EELS analysis.

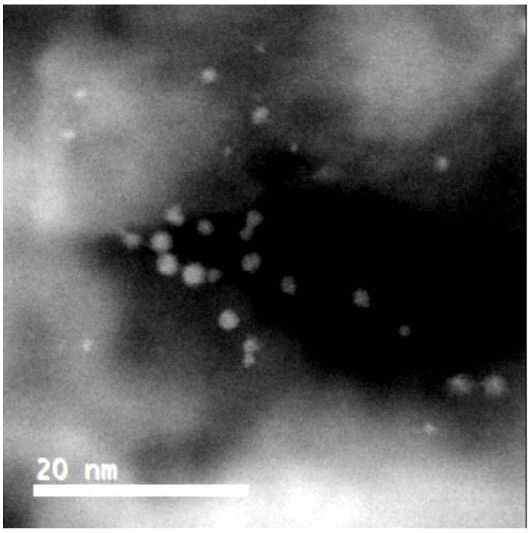

Supplementary Fig. 11 HAADF-STEM image of 0.1acacPt/C12A7.

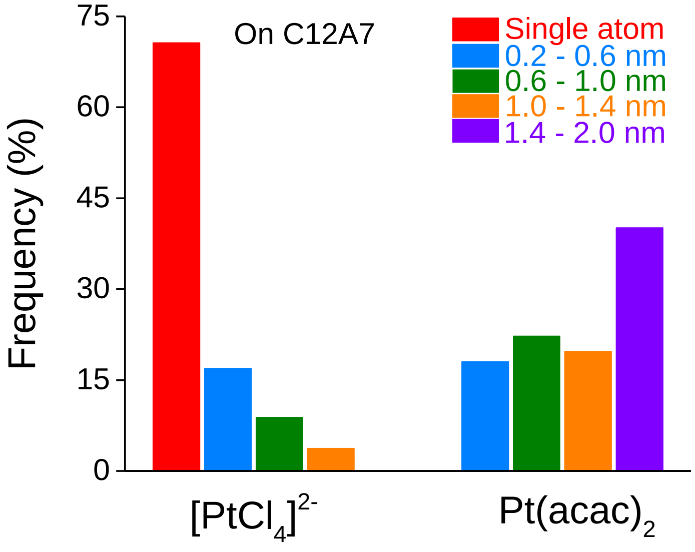

Supplementary Fig. 12 Histogram of size distributions of 0.1Pt/C12A7 with different Pt precursors (summarized from several HAADF-STEM images).

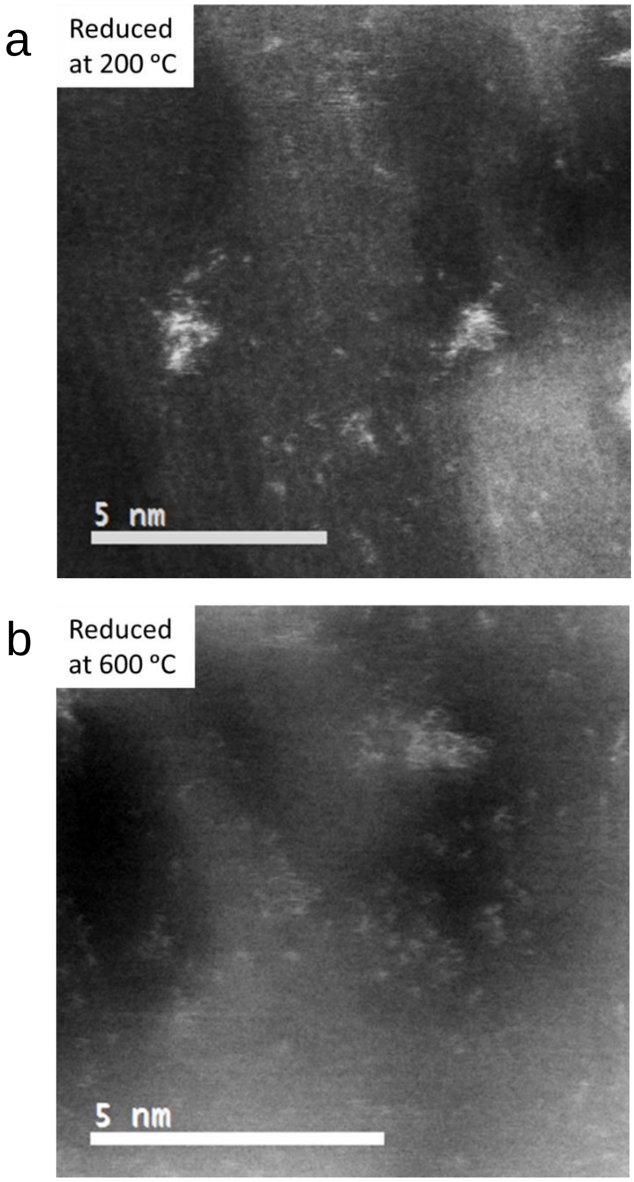

Supplementary Fig. 13 HAADF-STEM image of a) 0.1Pt/C12A7 and b) 0.1Pt/C12A7-R600.

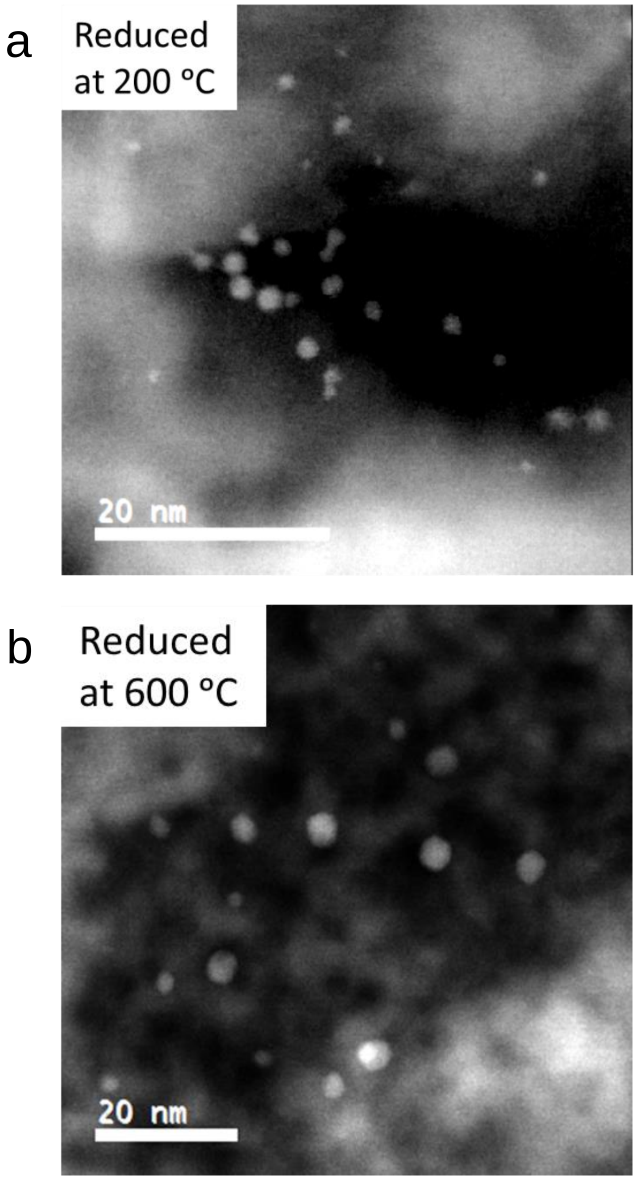

Supplementary Fig. 14 HAADF-STEM image of a) 0.1acacPt/C12A7 and b), 0.1acacPt/C12A7-R600.

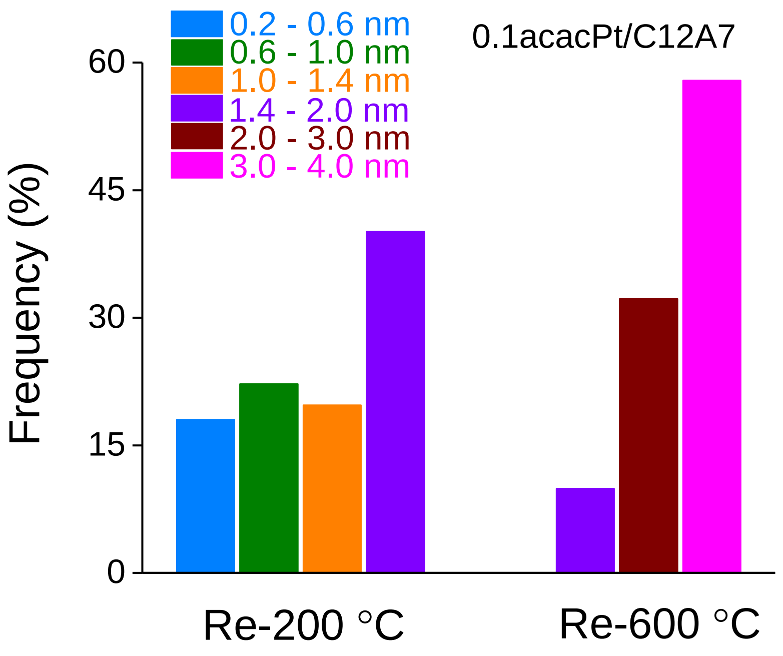

Supplementary Fig. 15 Histogram of size distributions of 0.1acacPt/C12A7 with different reduction temperature (summarized from HAADF-STEM images).

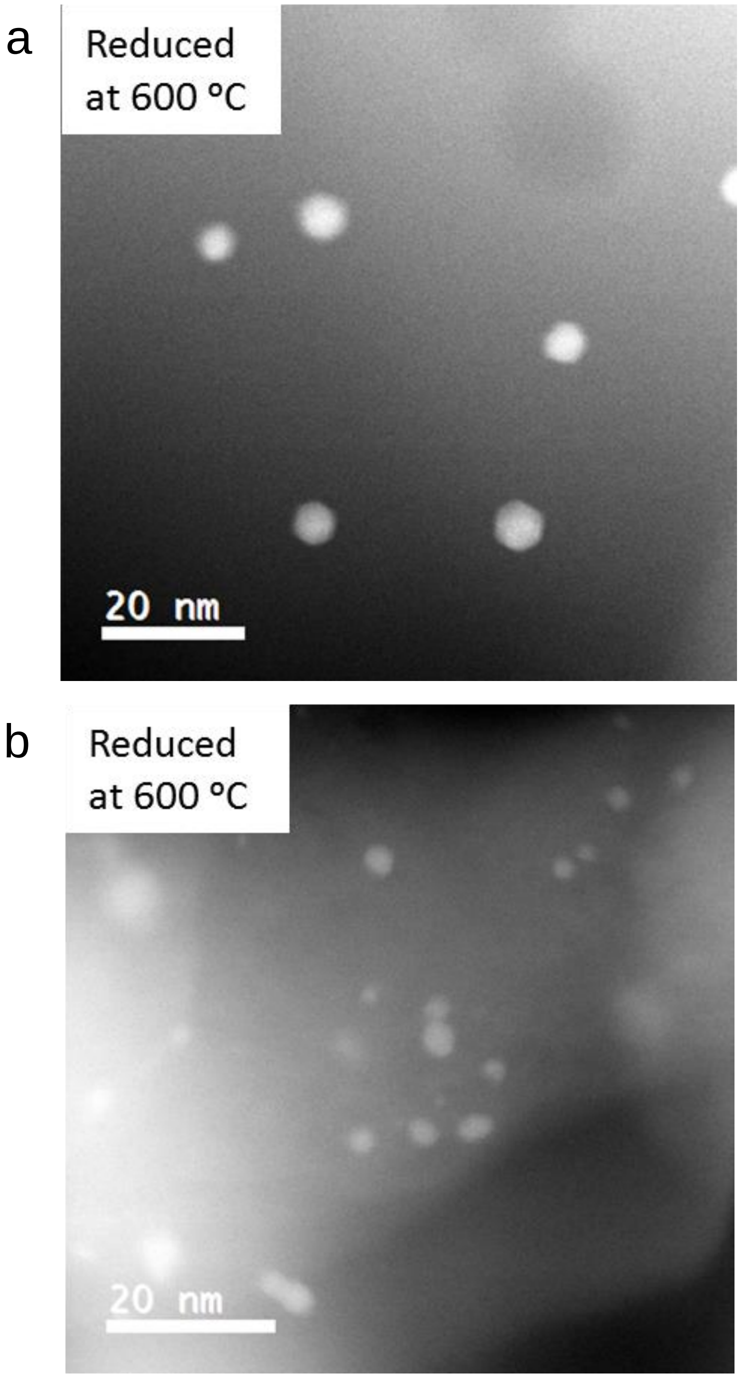

Supplementary Fig. 16 HAADF-STEM image of a) 0.1Pt/Al_2O_3-R600 and b) 0.1Pt/CaO-R600.

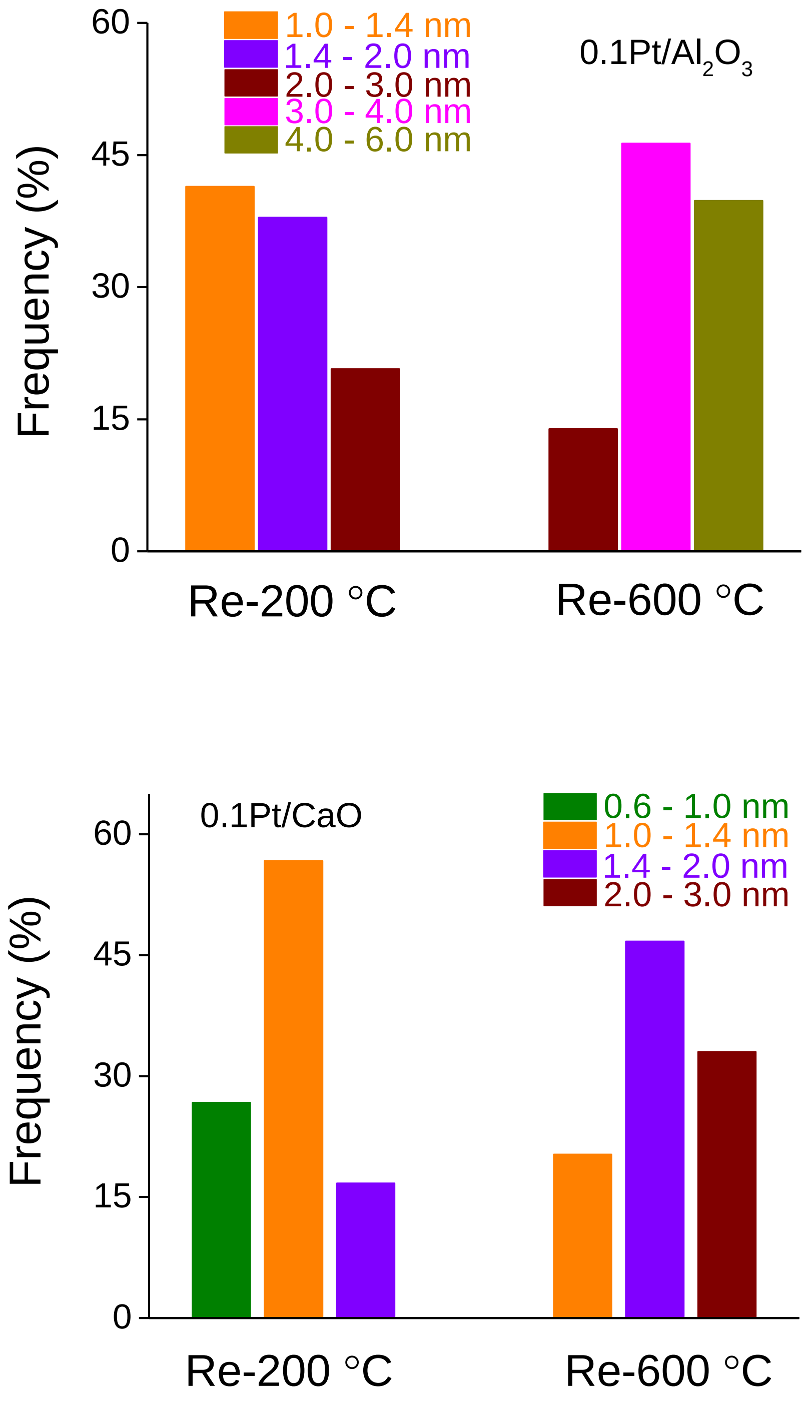

Supplementary Fig. 17 Histogram of size distributions of 0.1Pt/Al_2O_3 and 0.1Pt/CaO with different reduction temperature.

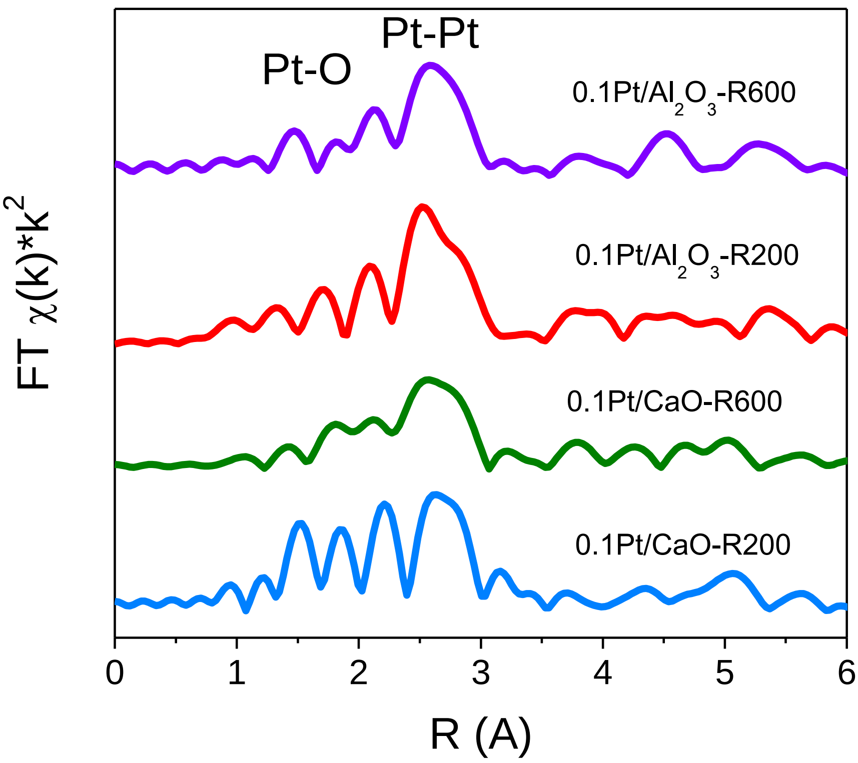

Supplementary Fig. 18 Pt K-edge EXAFS spectra in R space for 0.1Pt/Al_2O_3 and 0.1Pt/CaO with elevated reduction temperatures.

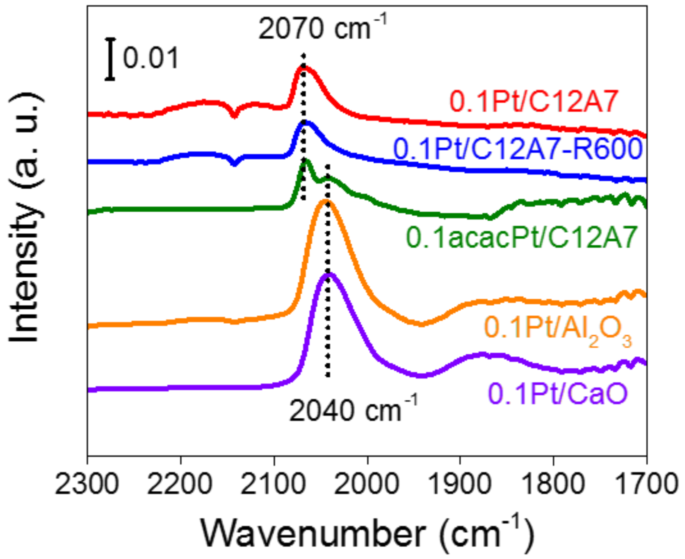

Supplementary Fig. 19 FTIR spectra of CO adsorption on 0.1Pt/C12A7, 0.1Pt/C12A7-R600, 0.1acacPt/C12A7, 0.1Pt/Al_2O_3 and 0.1Pt/CaO, respectively.

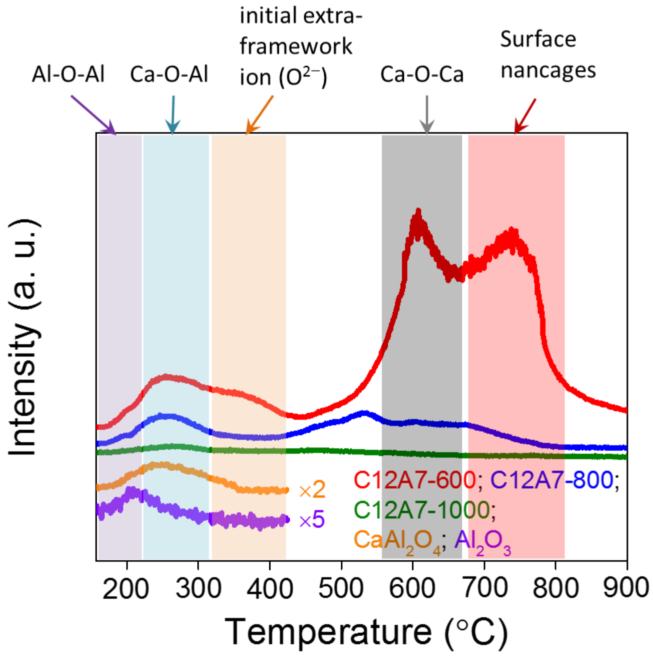

Supplementary Fig. 20 CO_2-TPD profiles of C12A7 prepared by different calcination temperature and Al_2O_3 and $CaAl_2O_4$ as referenced samples.

The peak at 220 °C can be ascribed to the oxygen site coordinated by two neighboring Al, which can be further proved by the CO_2 desorption from Al_2O_3 surface. The peak at 265 °C is mainly caused by the Ca-O-Al coordination unit. This is supported by the appearance of a broad CO_2 desorption peak ranging from 180 °C to 330 °C in $CaAl_2O_4$ which only contains Al-O-Al and Ca-O-Al units. Deducting the Al-O-Al peak at 220 °C, the rest peak at 265 °C comes from the CO_2 desorbed at Ca-O-Al units. The peak at 360 °C results from the initial extra-framework ion (O^{2-}) that merged with the cage wall according to our recent density of states (DOS) calculations results. This result demonstrates the existence of surface cavities.

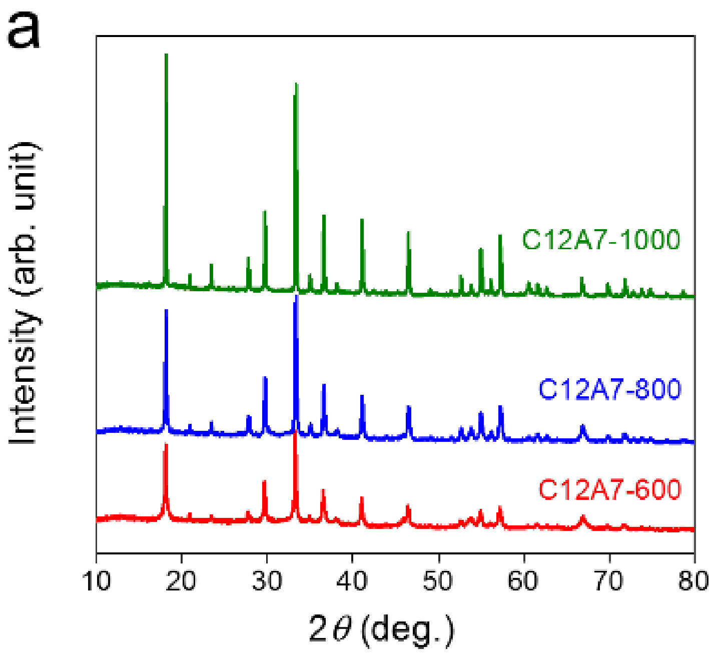

Supplementary Fig. 21 XRD patterns of C12A7 support prepared by different calcination temperature.

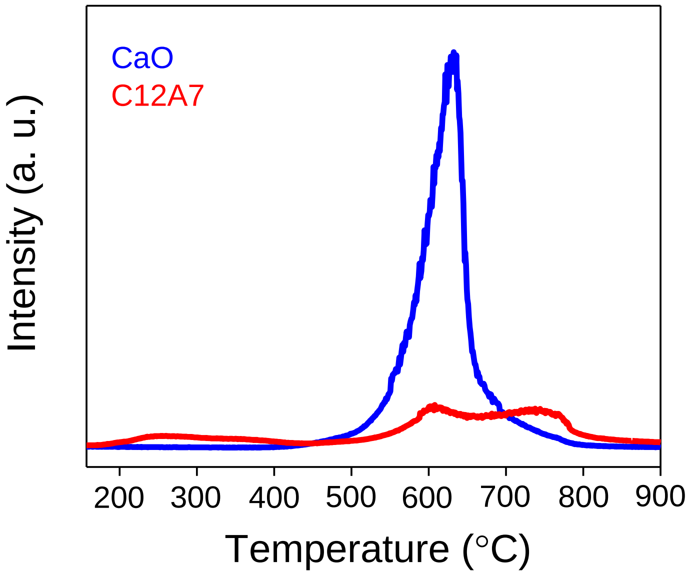

Supplementary Fig. 22 CO_2-TPD profiles of pure CaO and C12A7 respectively.

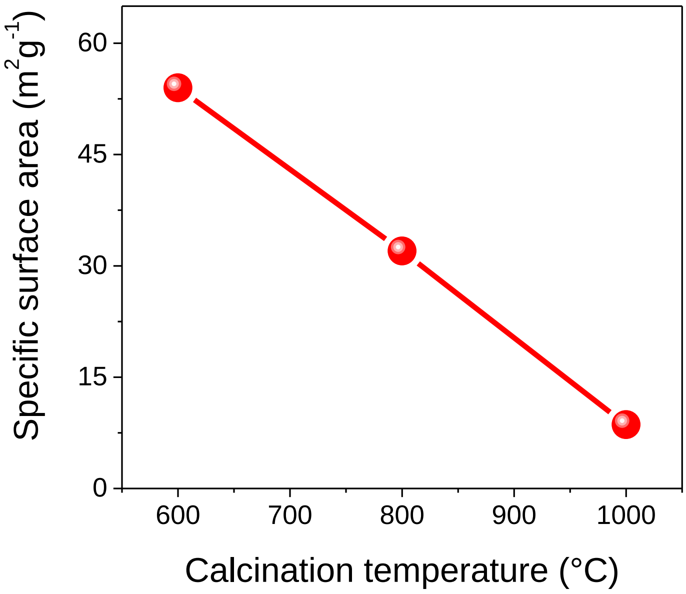

Supplementary Fig. 23 Corresponding specific surface area change of C12A7 support prepared by different calcination temperature.

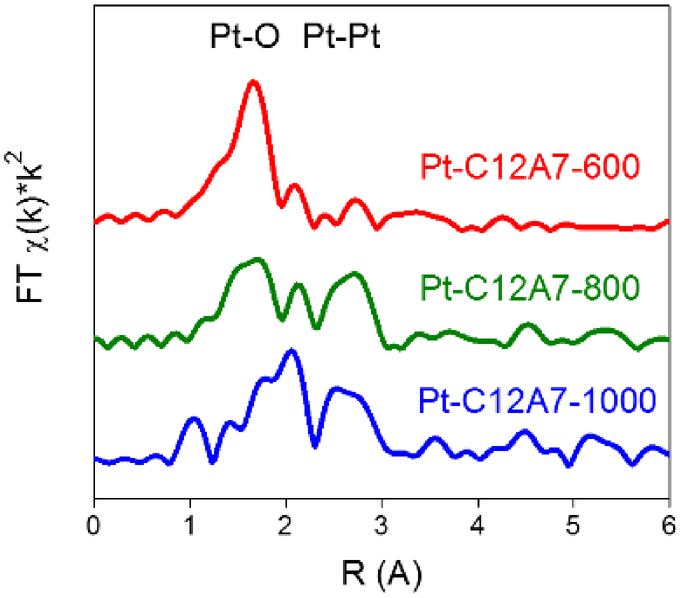

Supplementary Fig. 24 Pt K-edge EXAFS spectra in R space for 0.1Pt loaded on C12A7 support prepared by different calcination temperature.

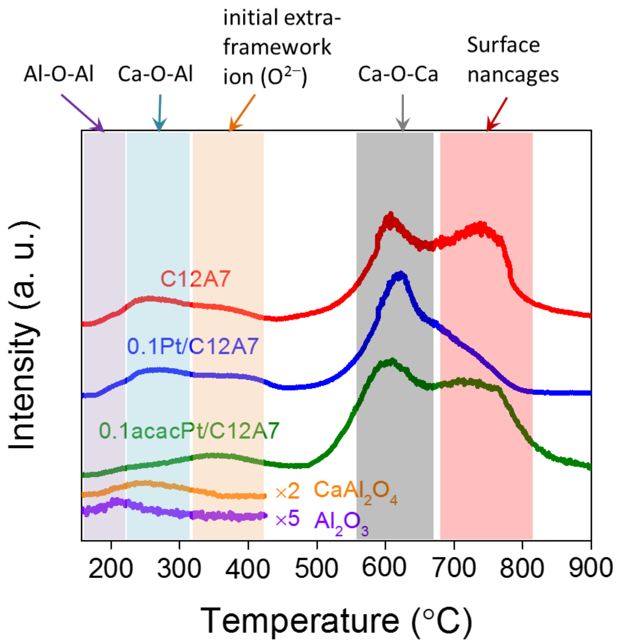

Supplementary Fig. 25 CO_2-TPD profiles of pure C12A7 and 0.1Pt loaded on C12A7 with different Pt precursors and Al_2O_3 and $CaAl_2O_4$ as referenced samples.

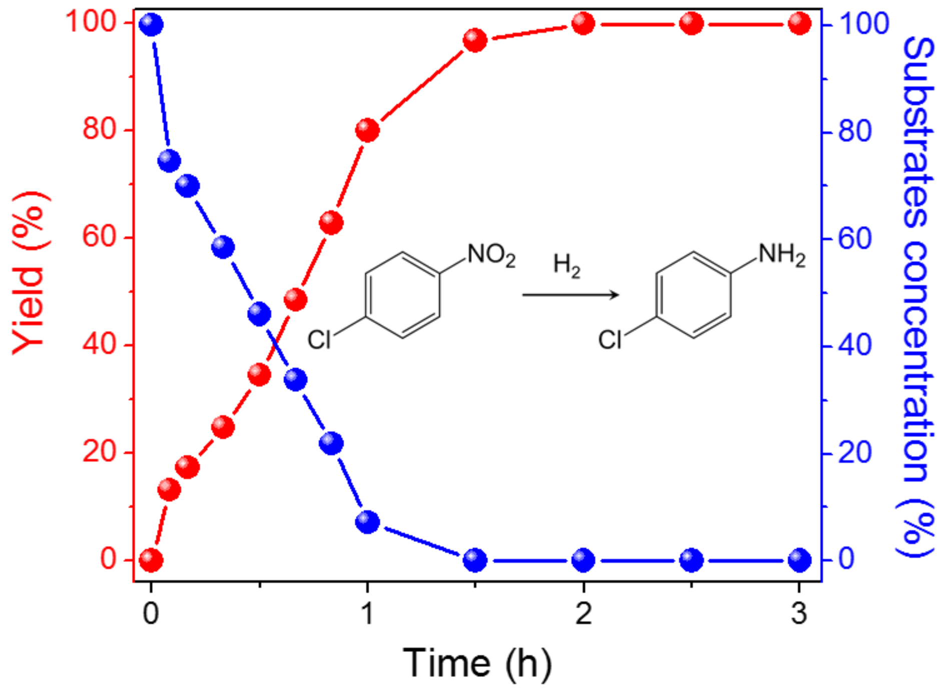

Supplementary Fig. 26 Kinetic studies of time-dependent catalysis over 0.1Pt/C12A7 towards the hydrogenation of 4-Chloronitrobenzene.

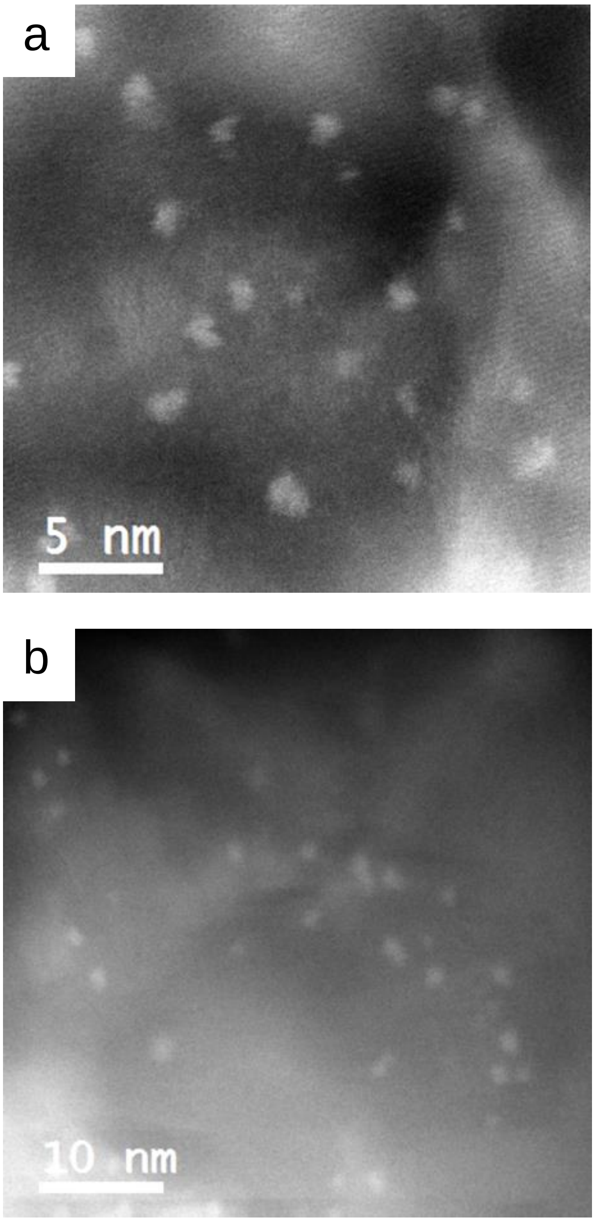

Supplementary Fig. 27 HAADF-STEM image of a) 0.5Pt/C12A7 and b) 2.0Pt/C12A7.

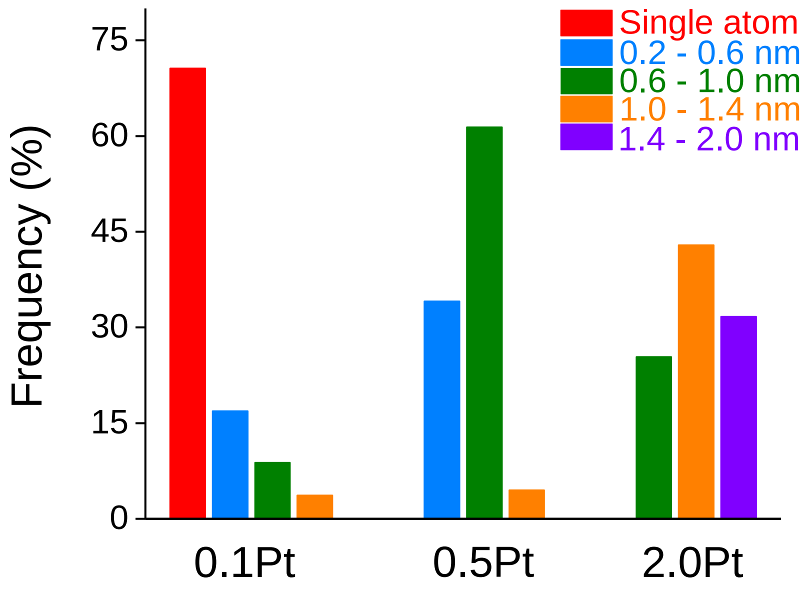

Supplementary Fig. 28 Histogram of size distributions of Pt/C12A7 with different Pt loading amount (summarized from HAADF-STEM images).

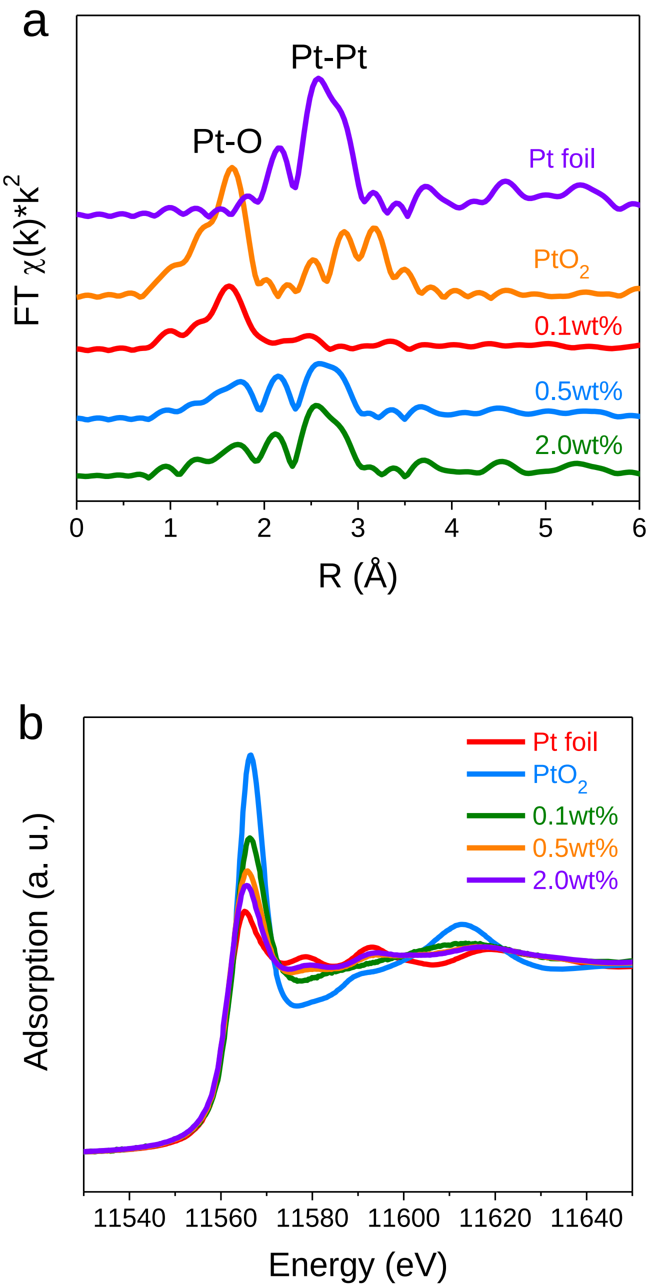

Supplementary Fig. 29 a) Pt K-edge EXAFS spectra in R space and b) the normalized XANES spectra at the Pt L3-edge of different loading amount of Pt species on C12A7.

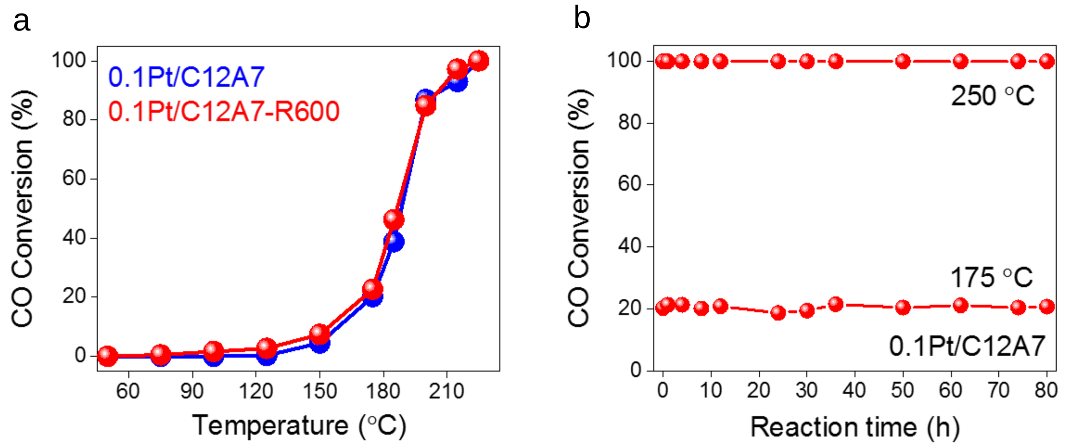

Supplementary Fig. 30 Evaluation of 0.1Pt/C12A7 catalysts in CO oxidation reaction. a. Conversion of CO from 50 to 230 °C over 0.1Pt/C12A7 and 0.1Pt/C12A7-R600 respectively. b. Time course for CO conversion at 175 °C and 250 °C over 0.1Pt/C12A7 catalyst.

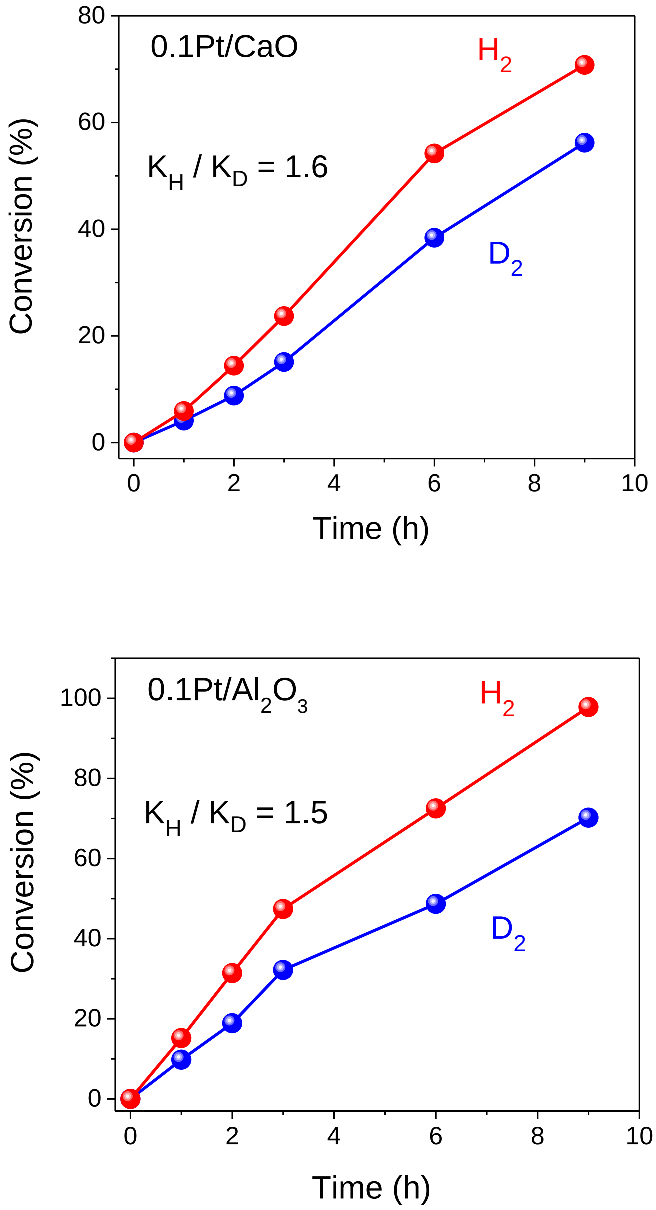

Supplementary Fig. 31 Primary isotope effect observed on 0.1Pt/Al_2O_3 and 0.1Pt/CaO in the hydrogenation of 4-Chloronitrobenzene.

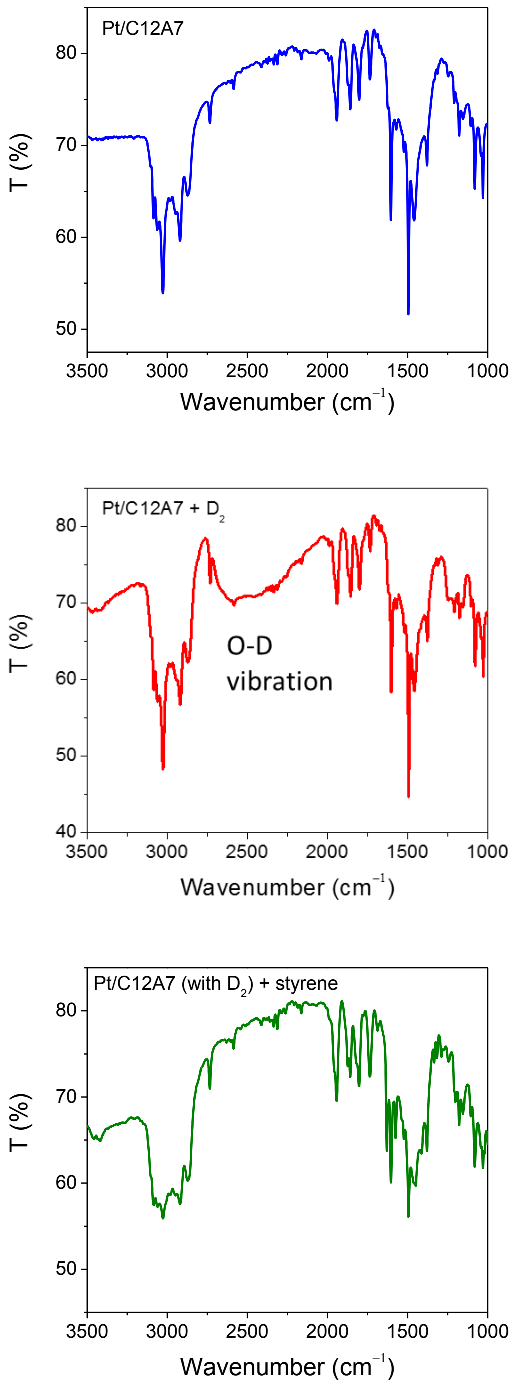

Supplementary Fig. 32 The FTIR spectrum of the 0.1Pt/C12A7 after exposure to D_2, showing O–D vibration. The vibration disappears after introduction of styrene.

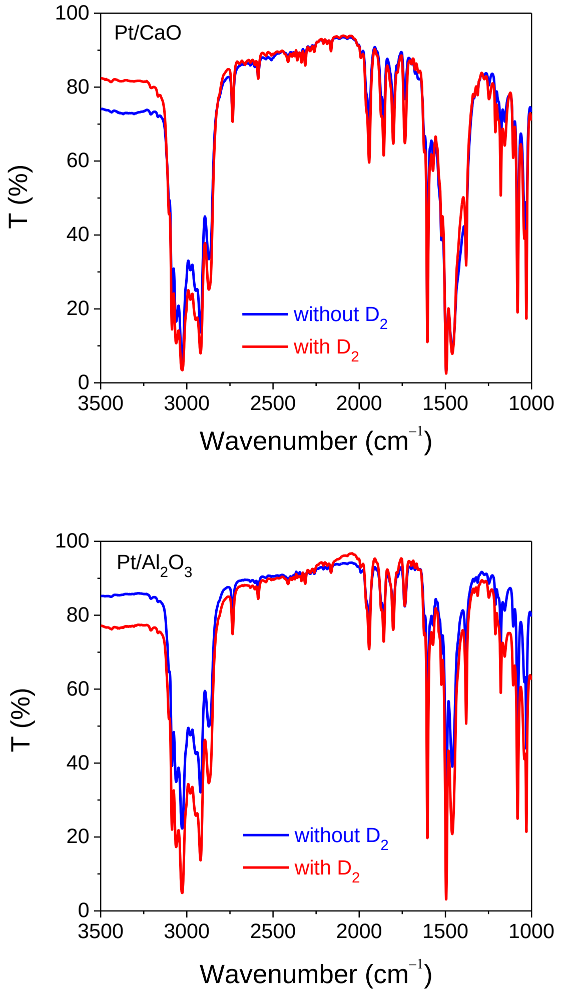

Supplementary Fig. 33 The FTIR spectra of $0.1Pt/Al_2O_3$ and 0.1Pt/CaO after exposure to D_2 without O–D vibration generated.

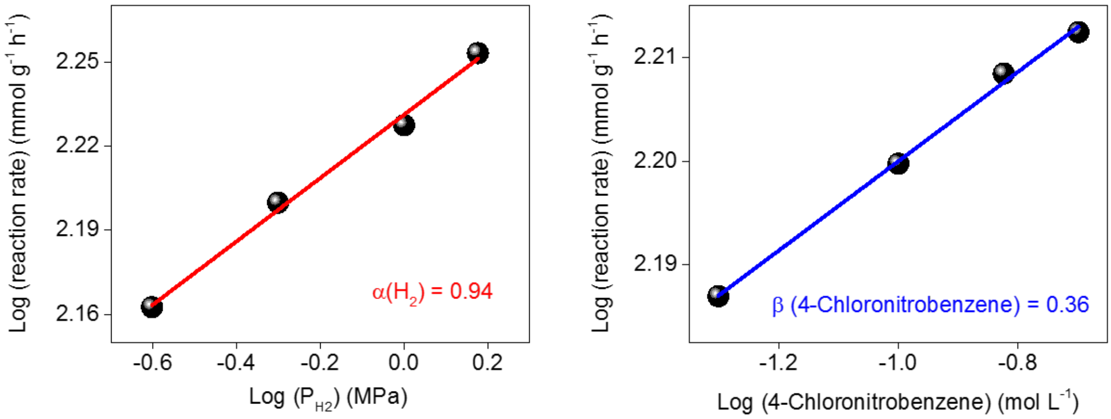

Supplementary Fig. 34 Dependence of reaction rate on partial pressures of H_2 and 4-chloronitrobenzene concentration.

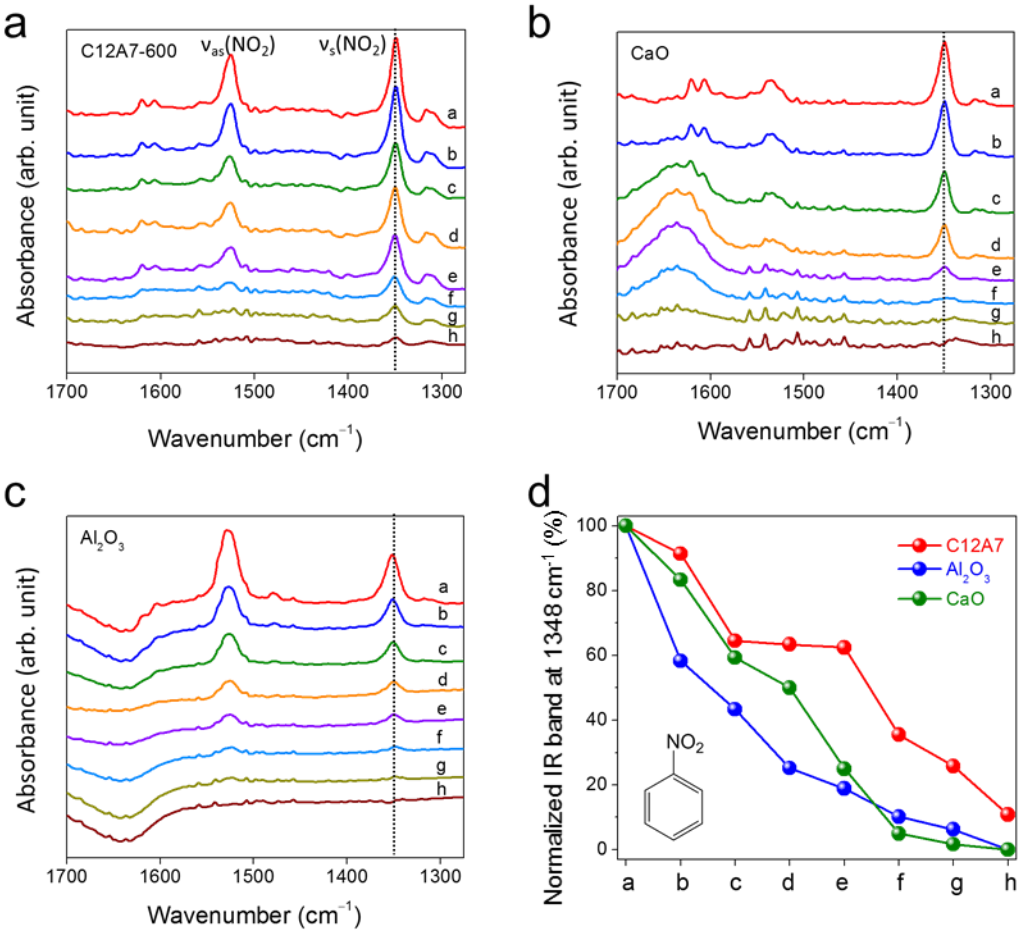

Supplementary Fig. 35 DRIFT spectra of the nitrobenzene adsorbed on a) C12A7, b) CaO and c) Al_2O_3 supports during the TPD process. Desorption conditions: (a) 1.3 Pa, (b) 10^{-1} Pa, (c) 10^{-2} Pa, (d) 10^{-3} Pa, (e) 10^{-4} Pa, (f) 40 °C, evac 30 min, (g) 70 °C, evac 30 min, (h) 100 °C, evac 30 min; d. Changes in FTIR band at 1348 cm^{-1} intensities during the TPD process.

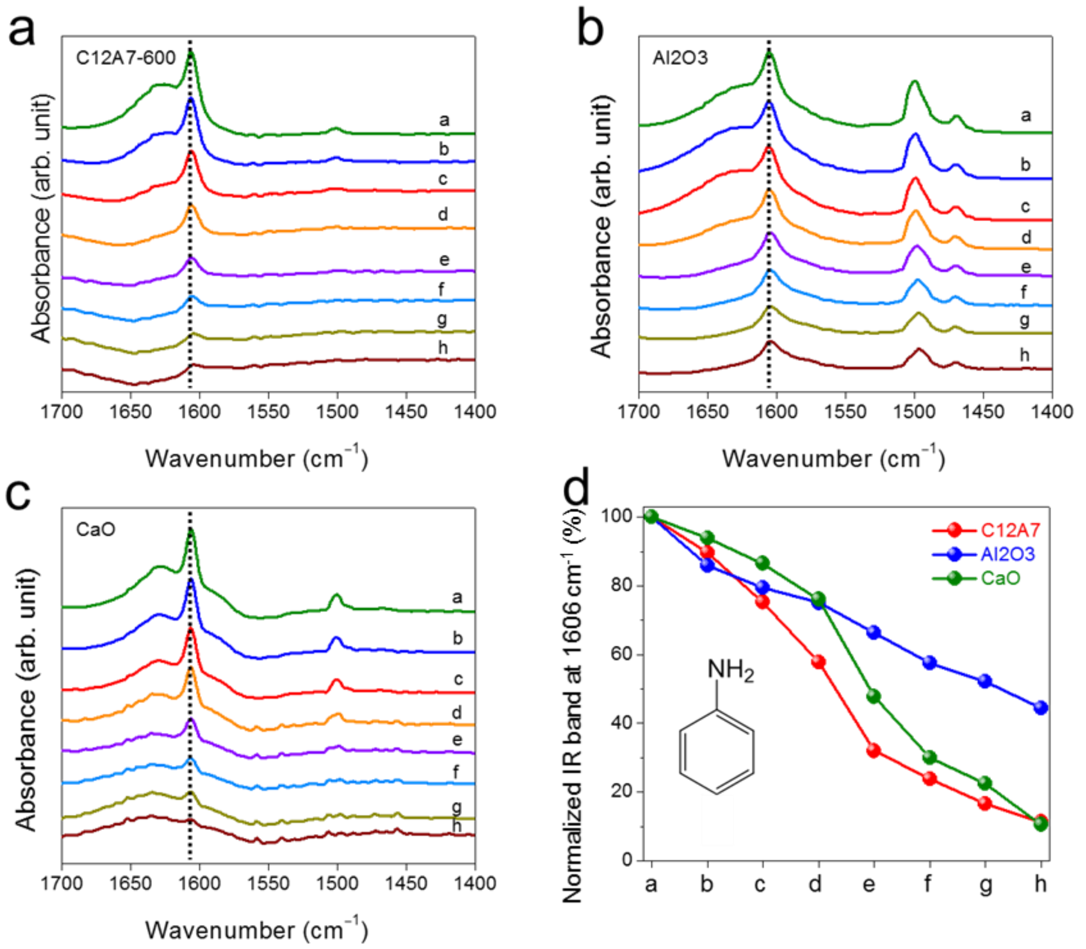

Supplementary Fig. 36 DRIFT spectra of the aniline adsorbed on a) C12A7, b) Al_2O_3 and c) CaO supports during the TPD process. measurement conditions: (a) 1.3 Pa, (b) 10^{-1} Pa, (c) 10^{-2} Pa, (d) 10^{-3} Pa, (e) 10^{-4} Pa, (f) 40 °C, evac 30 min, (g) 70 °C, evac 30 min, (h) 100 °C, evac 30 min; d. Changes in FTIR band at 1606 cm^{-1} intensities during the TPD process.

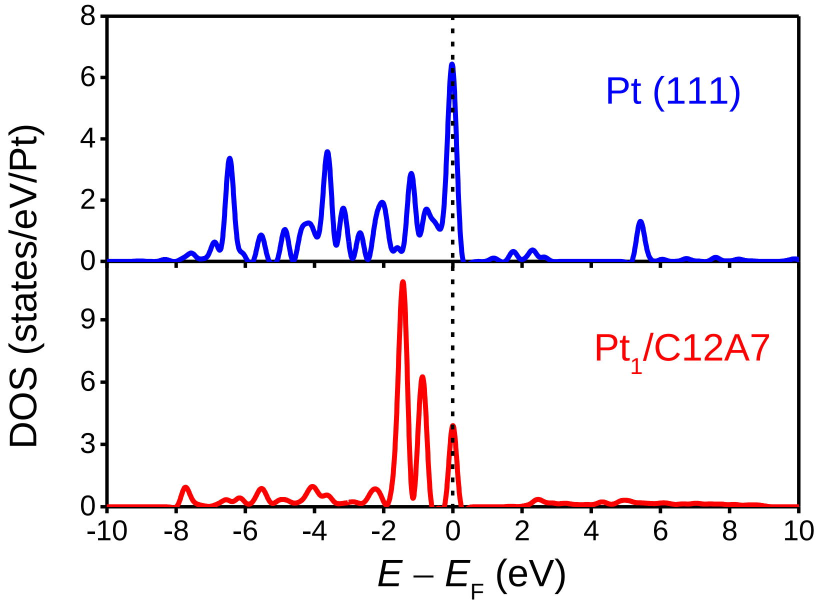

Supplementary Fig. 37 Projected Pt *d*-band densities of states (DOS). The *d*-band center (ε_d) is at –2.59 and –2.04 eV for Pt (111) surface and Pt_1/C12A7, respectively.

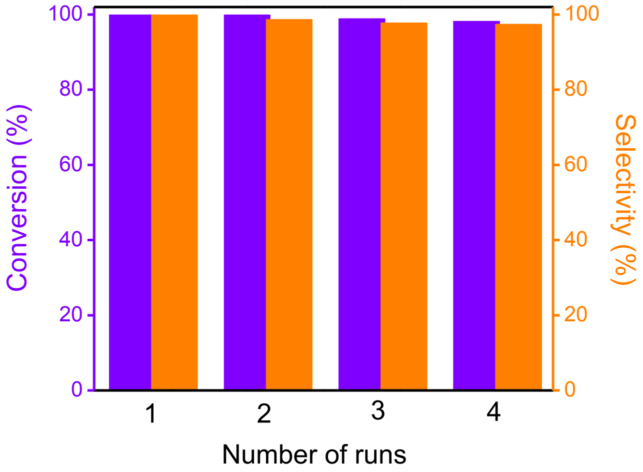

Supplementary Fig. 38 Cycle experiments using 0.1Pt/C12A7 as the catalyst. Reaction condition: 0.5 mmol 4-Chloronitrobenzene, 60 °C, 0.5 MPa H_2, 5 mg catalyst, 5 mL methanol.

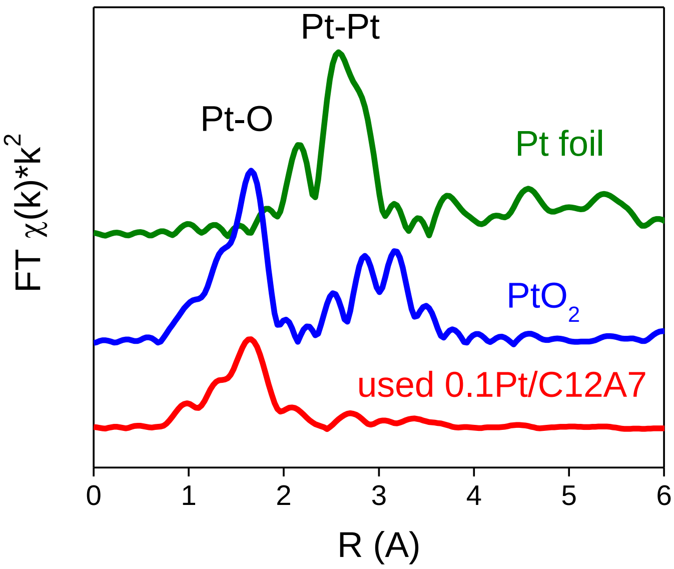

Supplementary Fig. 39 Pt K-edge EXAFS spectra in R space for the spent 0.1Pt/C12A7.

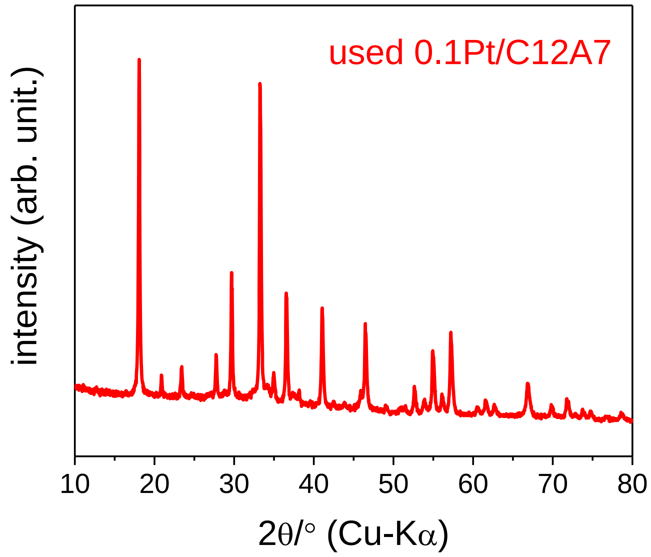

Supplementary Fig. 40 XRD pattern for 0.1Pt/C12A7 after the catalytic reaction.

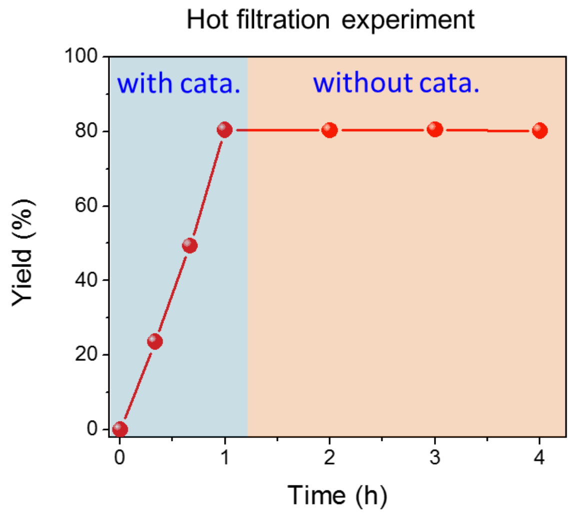

Supplementary Fig. 41 Hot filtration test of 0.1Pt/C12A7 for the hydrogenation of 4-Chloronitrobenzene. The hydrogenation reaction no longer proceeds after removal of the catalyst. Reaction conditions: 0.5 mmol 4-Chloronitrobenzene, 60 °C, 0.5 MPa H_2, 5 mg catalyst, 5 mL methanol.

Supplementary Table 1 Summary of Pt based catalysts with various supports and Pt precursors under different preparation conditions.

Entry	Support materials	Support calcination temperature (°C)	Pt precursor	Pt ratio (wt%)	Pt Reduction temperature (°C)	Representation in the main text
1	C12A7	600	$[PtCl_4]^{2-}$	0.12	200	0.1Pt/C12A7
2	Al_2O_3	---	$[PtCl_4]^{2-}$	0.13	200	0.1Pt/Al_2O_3
3	CaO	---	$[PtCl_4]^{2-}$	0.1	200	0.1Pt/CaO
4	C12A7	600	$[PtCl_4]^{2-}$	0.12	600	0.1Pt/C12A7-R600
5	Al_2O_3	---	$[PtCl_4]^{2-}$	0.13	600	0.1Pt/Al_2O_3-R600
6	CaO	---	$[PtCl_4]^{2-}$	0.1	600	0.1Pt/CaO-R600
7	C12A7	600	$[acac]_2Pt^{2+}$	0.11	200	0.1acacPt/C12A7
8	C12A7	600	$[acac]_2Pt^{2+}$	0.11	600	0.1acacPt/C12A7-R600
9	C12A7	800	$[PtCl_4]^{2-}$	0.11	200	0.1Pt/C12A7-800
10	C12A7	1000	$[PtCl_4]^{2-}$	0.15	200	0.1Pt/C12A7-1000
11	C12A7	600	$[PtCl_4]^{2-}$	0.32	200	0.3Pt/C12A7
12	C12A7	600	$[PtCl_4]^{2-}$	0.55	200	0.5Pt/C12A7
13	C12A7	600	$[PtCl_4]^{2-}$	0.97	200	1.0Pt/C12A7
14	C12A7	600	$[PtCl_4]^{2-}$	2.15	200	2.0Pt/C12A7

Supplementary Table 2 TPD-CO_2 profile of various compared samples

Sample	Surface area ($m^2 \cdot g^{-1}$)	Amount of basic site (μmol of $CO_2 \cdot g^{-1}$)				
		Al-O-Al	Ca-O-Al	[a]OH^-	[b]Ca-O-Ca	Surface truncated nanocages
C12A7	54	0.4	1.6	0.9	8.8	9.1
0.1Pt/C12A7	52	0.4	1.8	1.0	9.2	3.3

[a]Initial extra-framework ion that merged with the cage wall in the surface truncated nanocages. [b]Small amount of uncrystallized CaO species under low temperature (600 °C) calcination of hydrothermal obtained C12A7 precursor.

Supplementary Table 3 Chemoselective hydrogenation of 4-chloronitrobenzene on different Pt/C12A7 catalysts

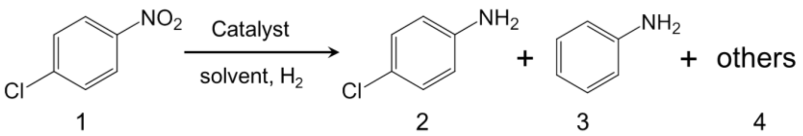

Entry	Catalyst	T (°C)	t (h)	Conv. (%)	Select. (%)		
					2	3	others
1	0.1Pt/C12A7	60	2	99.9	99.9	0	0
2	0.1Pt/C12A7	25	9	99.9	99.9	0	0
3	0.3Pt/C12A7	60	2	99.9	99.3	0.6	0
4	0.5Pt/C12A7	60	2	99.9	98.9	1.0	0
5	1.0Pt/C12A7	60	2	99.9	82.3	12.5	5.1
6	1.0Pt/C12A7	60	0.5	54.7	75.6	16.4	7.9
7	2.0Pt/C12A7	60	2	99.9	77.9	12.9	9.1
8	2.0Pt/C12A7	60	0.5	69.2	79.2	13.6	7.1
9	C12A7	60	9	---	---	---	---
10	Blank	60	9	---	---	---	---

Reaction condition: 0.5 mmol substrate, 5 mg catalyst, 5 ml methanol, 0.5 MPa H_2. Conversion (Conv.) and selectivity (Select.) were determined by GC and GCMS using n-hexadecane as an internal standard.

Supplementary Table 4 Summary of reported chemoselective hydrogenation of 4-chloronitrobenzene

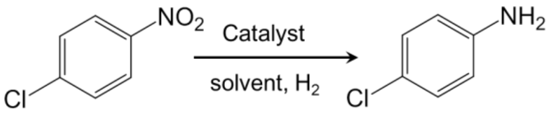

Catalyst	Condition	TOF (h^{-1})	Reference
**0.1Pt/C12A7**	**0.5 MPa H_2, 25 °C, 10 mins**	**8550**	**This work**
**0.1Pt/C12A7**	**0.5 MPa H_2, 40 °C, 5 mins**	**15715**	**This work**
**0.1Pt/C12A7**	**0.5 MPa H_2, 60 °C, 5 mins**	**25772**	**This work**
**0.1Pt/C12A7**	**0.5 MPa H_2, 60 °C, 120 mins**	**8125**	**This work**
Pt/RGO-$_{EG}$	1.0 MPa H_2, 40 °C, 120 mins	4577	*Carbon* **50**, 586 (2012)
Pt/CNF-P	1.0 MPa H_2, 25 °C, 120 mins	815	*Org. Lett.* **10**, 1601 (2008)
Pt/γ-Fe2O3	0.1 MPa H_2, 25 °C, 48 mins	3570	*J. Mol. Catal. A: Chem.***366**, 288 (2013)
KCC-1-PEI/Pt	1.0 MPa H_2, 25 °C, 480 mins	244	*J. Mater. Chem. A.***4**, 12416 (2016)
PtIPr$^{0.2}$	0.1 MPa H_2, 30 °C, 90 mins	317	*ChemCatChem.***6**, 87 (2014)
Pt NW	0.1 MPa H_2, 40 °C, 300 mins	15.6	*Chem. Eur. J.* **17**, 2763 (2011)
Pt/N-CNF-H	1.0 MPa H_2, 25 °C, 360 mins	212	*ChemCatChem.***3**, 1578 (2011)
Pt/PICP	0.1 MPa H_2, 25 °C, 60 mins	192	*Adv. Synth. Catal.***353**, 1260 (2011)

Supplementary Table 5 Summary of results reported for chemoselective hydrogenation of 3-chloronitrobenzene

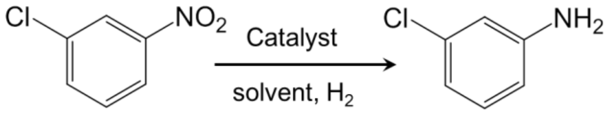

Catalyst	Condition	TOF (h^{-1})	Reference
**Pt/C12A7**	**0.5 MPa H_2, 25 °C, 10 mins**	**9211**	**This work**
**Pt/C12A7**	**0.5 MPa H_2, 40 °C, 5 mins**	**16720**	**This work**
**Pt/C12A7**	**0.5 MPa H_2, 60 °C, 5 mins**	**28822**	**This work**
**Pt/C12A7**	**0.5 MPa H_2, 60 °C, 100 mins**	**9750**	**This work**
Pt/TiO2	0.4 MPa H_2, 45 °C, 60 mins	131	*J. Am. Chem. Soc.* **130**, 8748 (2008)
Pt/γ-Fe2O3	0.1 MPa H_2, 25 °C, 72 mins	2352	*J. Mol. Catal. A: Chem.***366**, 288 (2013)
Pt/N-CNF-H	1.0 MPa H_2, 25 °C, 360 mins	215	*ChemCatChem.***3**, 1578 (2011)
Pt/PICP	0.1 MPa H_2, 25 °C, 60 mins	64	*Adv. Synth. Catal.***353**, 1260 (2011)

Supplementary Table 6 Summary of results reported for chemoselective hydrogenation of 2-chloronitrobenzene

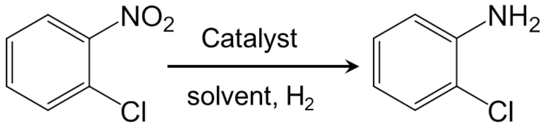

Catalyst	Condition	TOF (h^{-1})	Reference
**Pt/C12A7**	**0.5 MPa H_2, 25 °C, 10 mins**	**9688**	**This work**
**Pt/C12A7**	**0.5 MPa H_2, 40 °C, 5 mins**	**16081**	**This work**
**Pt/C12A7**	**0.5 MPa H_2, 60 °C, 5 mins**	**27947**	**This work**
**Pt/C12A7**	**0.5 MPa H_2, 60 °C, 120 mins**	**8811**	**This work**
Pt/FeO_x	0.3 MPa H_2, 40 °C, 60 mins	1218	*Nat. Commun.* **5**, 5634 (2014)
PtCo@NHPC	0.68 MPa H_2, 50 °C, 40 mins	422	*J. Am. Chem. Soc.* **138**, 11872 (2016)
Pt@NHPC	0.68 MPa H_2, 50 °C, 40 mins	209	*J. Am. Chem. Soc.* **138**, 11872 (2016)
Pt/RGO-$_{EG}$	1.0 MPa H_2, 40 °C, 120 mins	4162	*Carbon* **50**, 586 (2012)
Pt/γ-Fe2O3	0.1 MPa H_2, 25 °C, 60 mins	2568	*J. Mol. Catal. A: Chem.***366**, 288 (2013)
Pt/N-CNF-H	1.0 MPa H_2, 25 °C, 360 mins	319	*ChemCatChem.***3**, 1578 (2011)
